# New insight into the CNC-bZIP member, NFE2L3, in human diseases

**DOI:** 10.3389/fcell.2024.1430486

**Published:** 2024-07-23

**Authors:** Guanghui Xiong, Jie Li, Fuli Yao, Fang Yang, Yuancai Xiang

**Affiliations:** ^1^ Department of Biochemistry and Molecular Biology, School of Basic Medical Sciences, Southwest Medical University, Luzhou, Sichuan, China; ^2^ Department of Children Rehabilitation, Maternal and Child Health Hospital of Jintang County, Chendu, Sichuan, China; ^3^ Department of Anaesthesia, The Affiliated Hospital, Southwest Medical University, Luzhou, Sichuan, China; ^4^ Department of Physiology, School of Basic Medical Sciences, Southwest Medical University, Luzhou, Sichuan, China; ^5^ Department of Pathophysiology, College of High Altitude Military Medicine, Third Military Medical University (Army Medical University), Chongqing, China

**Keywords:** cancer, homoeostasis, stress, CNC-bZIP, NFE2L3/NRF3, post-transcriptional modification, transcriptional regulation

## Abstract

Nuclear factor erythroid 2 (NF-E2)-related factor 3 (NFE2L3), a member of the CNC-bZIP subfamily and widely found in a variety of tissues, is an endoplasmic reticulum (ER) membrane-anchored transcription factor that can be released from the ER and moved into the nucleus to bind the promoter region to regulate a series of target genes involved in antioxidant, inflammatory responses, and cell cycle regulation in response to extracellular or intracellular stress. Recent research, particularly in the past 5 years, has shed light on NFE2L3’s participation in diverse biological processes, including cell differentiation, inflammatory responses, lipid homeostasis, immune responses, and tumor growth. Notably, NFE2L3 has been identified as a key player in the development and prognosis of multiple cancers including colorectal cancer, thyroid cancer, breast cancer, hepatocellular carcinoma, gastric cancer, renal cancer, bladder cancer, esophageal squamous cell carcinoma, T cell lymphoblastic lymphoma, pancreatic cancer, and squamous cell carcinoma. Furthermore, research has linked NFE2L3 to other cancers such as lung adenocarcinoma, malignant pleural mesothelioma, ovarian cancer, glioblastoma multiforme, and laryngeal carcinoma, indicating its potential as a target for innovative cancer treatment approaches. Therefore, to gain a better understanding of the role of NFE2L3 in disease, this review offers insights into the discovery, structure, function, and recent advancements in the study of NFE2L3 to lay the groundwork for the development of NFE2L3-targeted cancer therapies.

## 1 Introduction

Cells respond to changes in their internal and external environments by initiating a series of biological reactions to preserve their stability and normal physiological functions, known as cellular stress. This form of stress can manifest in different ways, including oxidative stress, heat, hypoxia, endoplasmic reticulum (ER) stress, and genotoxic stress. Oxidative stress is a cellular stress reaction caused by an increase in cellular oxidative damage due to various factors, such as environmental changes, drug exposure, and metabolic abnormalities (Sies, 2015). This stress is primarily induced by oxygen free radicals such as superoxide anions, hydroxyl free radicals, and hydrogen peroxide. Prolonged oxidative stress can lead to the destruction of biological macromolecules, resulting in cellular dysfunction and death. Numerous studies have demonstrated that genes from the cap‘n’collar (CNC) -basic region leucine zipper (bZIP) subfamily act as a critical molecular switch for cells to counter intracellular oxidative stress (Bathish et al., 2022; Liu et al., 2023; Hu et al., 2024). These transcription factors are able to bind to the antioxidant or electrophile response element (ARE or EpRE) site in the promoter region of genes that code for anti-oxidative enzymes (Zhang and Xiang, 2016). The CNC-bZIP subfamily comprises six members in vertebrates, including nuclear factor-erythroid 2 (NF-E2) p45, NF-E2-related factor 1 (NFE2L1), NFE2L2, NFE2L3, BTB domain and CNC homolog 1 (BACH1), and BACH2, each of which plays key roles in various cellular functions, including proliferation, apoptosis, inflammatory response, embryonic development, and metabolic regulation (Yang et al., 2020; Cirone and D’Orazi, 2022; Waku and Kobayashi, 2021; Zhou et al., 2016).

**FIGURE 1:**
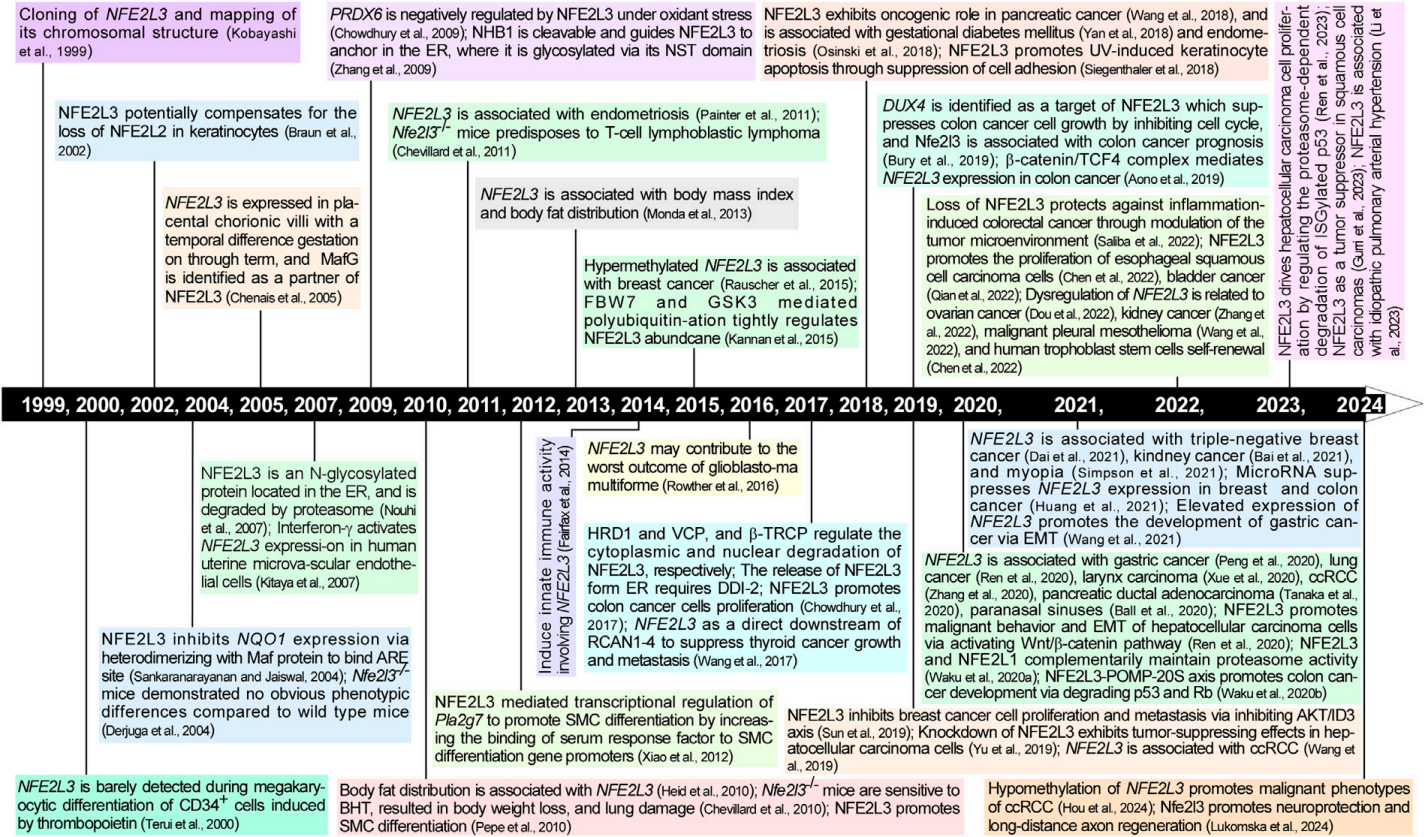
Timeline of research progression in NFE2L3 from 1999 to 2024. The timeline represents a major history of scientific research on NFE2L3 in the last two and half decades. Further details are provided in the text.

Compared with the well-known CNC-bZIP members NFE2L1 and NFE2L2, less attention has been paid to NFE2L3. However, recent research, particularly in the past 5 years, has shed light on NFE2L3’s participation in diverse biological processes including cell differentiation, inflammatory responses, oxidative stress, lipid homeostasis, transcription activation, immune response, and tumor growth (Figure 1). Notably, NFE2L3 has been identified as a key player in the development and prognosis of multiple cancer types, including colorectal (Waku and Kobayashi, 2021), liver (Ren Y. et al., 2020), thyroid (Wang et al., 2017), pancreatic (Wang et al., 2018), and renal cancers (Wang et al., 2019; Zhang et al., 2022). Furthermore, studies have linked *NFE2L3* to other cancers such as lung adenocarcinoma (Ren J. et al., 2020), malignant pleural mesothelioma (Wang et al., 2022), and ovarian cancer (Dou et al., 2022), indicating its potential as a target for innovative cancer treatment approaches. Therefore, to gain a better understanding of the role of NFE2L3, this review offers insights into the discovery, structure, function, and recent advancements in the study of NFE2L3 to lay the groundwork for the development of NFE2L3-targeted therapies for cancer.

## 2 Discovery, distribution, and sequence structure of NFE2L3

### 2.1 Discovery of NFE2L3

In the 1980s, the discovery of homeobox (Hox) gene clusters, HoxA, HoxB, HoxC, and HoxD, confirmed their critical role in early embryonic development in *Drosophila* (McGinnis and Krumlauf, 1992). These gene clusters encode a series of transcription factors essential for biological processes, such as embryonic development, body axis formation, and cell differentiation (Du and Taylor, 2015). Thereafter, the genes within these clusters have been extensively studied. Notably, cDNA encoding NF-E2 p45 (Chan and Kan, 1993a), NFE2L1 (Chan and Kan, 1993b), and NFE2L2 (Moi et al., 1994) have been found near the HoxC, HoxB and HoxD clusters, respectively. In 1999, Kobayashi et al. (1999) found an expressed sequence tag (EST) clone (668 bp) located near HoxA in The Institute for Genomic Research Human database (GenBank accession number THC181377); the encoded protein was highly homologous to the C-terminal amino acid sequence of NFE2L1. They then obtained a fragment that matched the EST clone in HeLa cell genomic DNA. To obtain the full-length cDNA, the human placental cDNA library was screened using PCR, and four positive overlapping phage clones were isolated. Furthermore, domain structure analysis showed that these newly cloned cDNAs contained CNC and bZIP domain, and had high homology with NF-E2 p45, NFE2L1, and NFE2L2. Therefore, this new member of the CNC subfamily is called nuclear factor-erythroid two related factor 3 (NRF3 or NFE2L3) (Kobayashi et al., 1999). Since then, the structure and function of NFE2L3 have gradually been unveiled with advancements in NFE2L3 research (Figure 1).

### 2.2 Distribution and cell localization of NFE2L3

Fluorescence *in situ* hybridization experiments have identified the human *NFE2L3* gene’s location on chromosome seven p15-p14, with widespread expression across multiple tissues (Kobayashi et al., 1999). Notably, *NFE2L3* displays low expression levels in most normal tissues but exhibits increased expression in specific organs such as the placenta (Kobayashi et al., 1999). Additionally, elevated *NFE2L3* expression has been detected in B cells, monocytes, placental trophoblasts, and tumor cells (Kobayashi et al., 1999; Chenais et al., 2005). Within cells, the full-length glycosylated isoform NFE2L3A is localized in the ER, while the full-length non-glycosylated isoform NFE2L3B is found in the cytosol. In contrast, the truncated isoform NFE2L3C is released from the ER and translocated to the nucleus to exert its transcriptional regulatory functions (Nouhi et al., 2007).

### 2.3 Domain distribution and their role for NFE2L3

**FIGURE 2:**
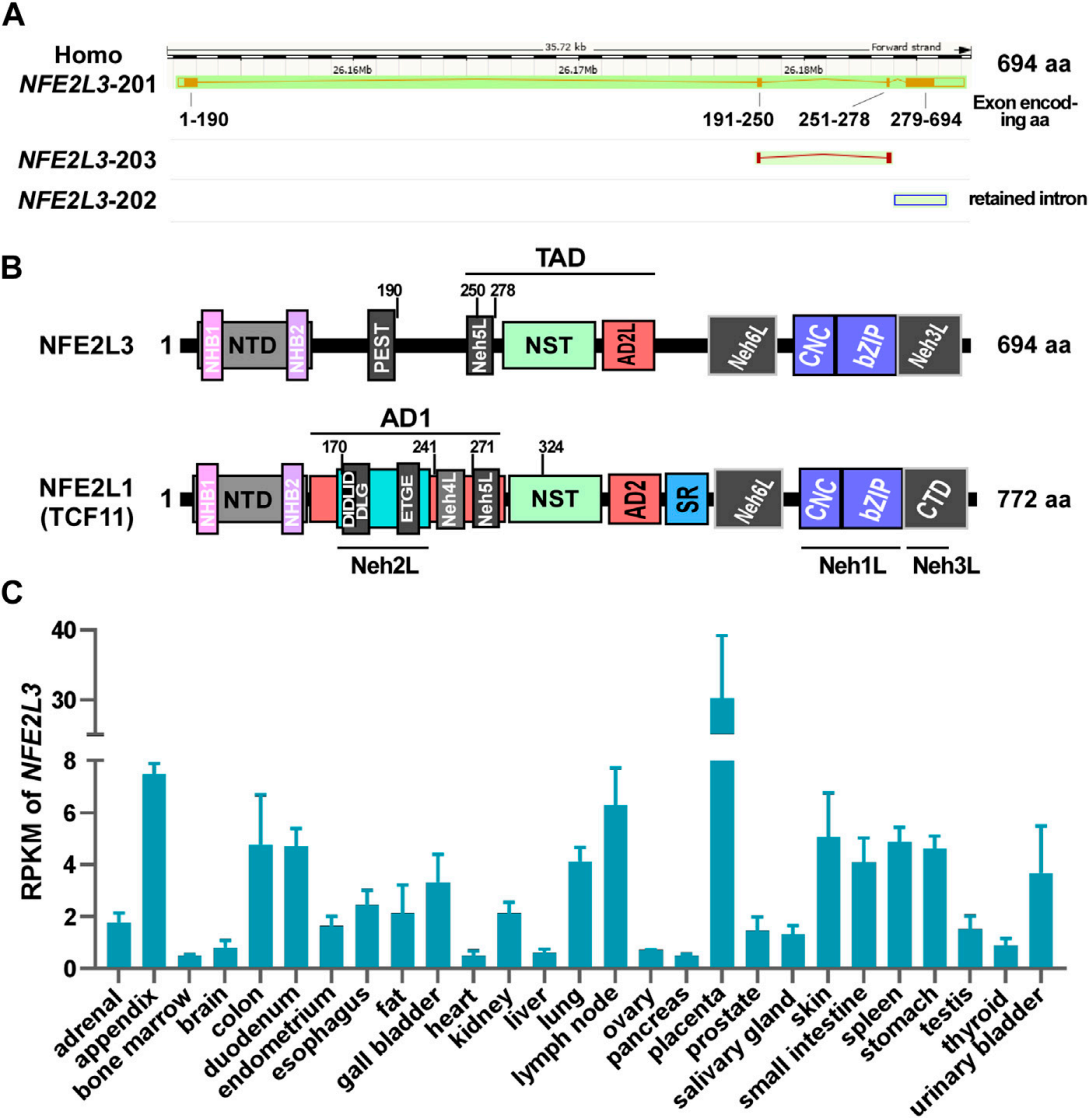
The detailed information of NFE2L3 and NFE2L1. **(A)** The genomic and transcriptional information of human *NFE2L3* were obtained from Ensembl genome browser. **(B)** The domain distribution of human NFE2L3 and NFE2L1 (TCF11). The detailed amino acid information of Nfe2L3 domains is provided in Table 1 **(C)** The reads per kilobase per million mapped reads (RPKM) of *NFE2L3* in different human tissues, which was obtained from GenBank database. AD1/2: acidic domain 1 or 2; bZIP: basic-leucine zipper; CNC: cap ‘n’ collar; CTD: C-terminal domain; Neh L: Neh-like; NHB1/2: N-terminal homology box 1 or 2; NST: Asn/Ser/Thr-rich; NTD: N-terminal domain; SR: serine-repeat.

Human NFE2L3 comprises of 694 amino acids, whereas mouse NFE2L3 is composed of 660 amino acids (Kobayashi et al., 1999). As a member of the CNC-bZIP subfamily, the structural domains of NFE2L3 exhibit high similarities to those of NFE2L1 and possess typical domains, including an N-terminal domain (NTD), a transactivation domain (TAD), and a DNA-binding domain (Figures 2A, B). Sequence alignment result revealed that human NFE2L3 consists of seven major domains: NTD (1–146 aa), Pro/Glu/Ser/Thr-rich sequence (PEST, 161–173 aa), TAD (233–431 aa), Nrf2-ECH homology 6-like (Neh6L, 432–535 aa), CNC (536–579 aa), bZIP (580–631 aa), and Neh3L (632–683 aa) (Zhang et al., 2009). These domains play crucial roles in the regulation of NFE2L3 activation, transcription activity, and degradation, with detailed information provided in Table 1.

**TABLE 1:** Protein functional domains of NFE2L3.

Domain	Abbreviation	Residues (aa)		Functional role	References
		Human	Mouse		
N-terminal domain	NTD	1–146	1–126	Negative regulates NFE2L3 activity	Chenais et al. (2005), Zhang et al. (2009)
N-terminal homology box 1	NHB1	13–32	12–31	This domain is part of a tripartite signal peptide sequence transmembrane region	Zhang et al. (2009)
N-terminal homology box 2	NHB2	90–114	76–100	Controlled the proteolytic processing of NFE2L3 into cleavage products	Zhang et al. (2009), Chowdhury et al. (2017)
Pro/Glu/Ser/Thr-rich	PEST	161–173	137–152	Contributes to protein rapid turnover, and negatively regulates NFE2L3 activity	Nouhi et al. (2007), Zhang et al. (2009)
Transactivation domain	TAD	233–431	211–400	Activate transcription	Zhang et al. (2009)
Neh5L	Neh5L	233–279	211–256	Highly conserved domains; a part of the TAD.	Zhang et al. (2009)
Asn/Ser/Thr-rich	NST	281–377	258–350	A potential glycosylation domain; a part of the TAD; this domain possesses potential a transcriptional activation function	Zhang et al. (2009)
Acidic Domain 2 like	AD2L	378–431	351–400	Highly conserved domains; a part of the TAD.	Chenais et al. (2005)
Neh6-like	Neh6L	432–535	401–498	Highly conserved domains; this domain exhibits a transcriptional activation role	Chenais et al. (2005)
Cap ‘n’ Collar	CNC	536–579	499–542	Highly conserved domain; deletion both CNC and bZIP increases transactivation	Chenais et al. (2005), Zhang et al. (2009)
Basic-leucine zipper	bZIP	580–631	543–604	DNA binding domain; deletion both CNC and bZIP increases transactivation	Chenais et al. (2005), Zhang et al. (2009)
Neh3-like	Neh3L	632–683	605–646	Highly conserved domains	Zhang et al. (2009)
Basic c-tail	BCT	684–694	647–660	Conserved domains between NFE2L3 and NFE2L1	Zhang et al. (2009)

## 3 Expression and regulation of NFE2L3

The discovery of NFE2L3 has led researchers to uncover its structure and function, generating interest in its associated regulatory mechanisms. Recent studies have suggested that the regulation of NFE2L3 mainly occurs at the transcriptional, post-transcriptional, and post-translational levels.

### 3.1 Transcriptional and post-transcriptional regulation of *NFE2L3*

A search of the Ensembl database revealed that human *NFE2L3* contains four exons that generating three transcripts (two protein-coding sequences and one retained intron sequence; Figure 2A), with the highest mRNA levels observed in embryos in the GenBank database (Figure 2C). Notably, in a study by Chenais et al. (2005), the mRNA and protein levels of NFE2L3 in choriocarcinoma JAR cells could be significantly increased in the treatment of TNF-α. Further, they found that TNF-α can promote *NFE2L3* transcription in colon adenocarcinoma through activating transcription factor p65 (RELA) which can bind to the first intron of *NFE2L3* (Bury et al., 2019). In addition, a similar work showed that the β-catenin/transcription factor 4 (TCF4) complex can bind to the TCF recognition element (CTTTGAA, Wnt responsive element, WRE site) within the first intron region of *NFE2L3* to promote its transcription (Aono et al., 2019). In addition to these direct regulations of the transcriptional process of *NFE2L3*, miRNAs are also important factors in regulating *NFE2L3* expression at the post-transcriptional level. For example, a luciferase reporter activity assay revealed that miR-1246 (Dai et al., 2021) and miR-23b-3p inhibit the expression of *NFE2L3* (Huang et al., 2021). Additionally, factors such as keratinocyte growth factor (KGF) (Braun et al., 2002), interferon-γ (Kitaya et al., 2007), and calcineurin 1-subtype 4 (RCAN1-4) (Wang et al., 2017) have been shown to influence *NFE2L3* expression, although the underlying mechanisms remain unknown.

### 3.2 Post-translational modification of NFE2L3

Post-translational modifications (PTMs) increase the functional diversity of the proteome by covalently attaching functional groups or proteins to protein molecules. The modifications included phosphorylation, glycosylation, ubiquitination, nitrosylation, methylation, acetylation, and lipidation. These modifications play a critical role in various aspects of both normal cell biology and pathogenesis (Ramazi and Zahiri, 2021; Peng et al., 2023). However, current studies have only revealed glycosylation, ubiquitination, and phosphorylation as the PTMs of NFE2L3, which was shown as follows.

#### 3.2.1 Glycosylation

Glycosylation is a common post-translational modification of proteins that involves the transfer of sugars to proteins or specific amino acid residues via glycosyltransferases to form glycosidic bonds. In a study by Nouhi et al. (2007), NFE2L3 has three main isoforms (NFE2L3A, NFE2L3B, and NFE2L3C). When treated with deglycosylase, isoform A was disappeared, leading to an increase in the abundance of isoform B, whereas that of isoform C remained unchanged. These findings suggest that the ER located isoform A is glycosylated, isoform B is non-glycosylated, and isoform C may be a processed non-glycosylated protein. This conclusion is supported by Zhang et al. (2009), who further demonstrated that glycosylation inhibits the transcriptional activity of NFE2L3, and speculated that isoform B may represent a glycosylated cleaved protein or deglycosylated protein. Overall, among NFE2L3 PTMs, glycosylation appears to be a crucial factor to modulate the generation of multiple isoforms with varying activities.

#### 3.2.2 Ubiquitination

Ubiquitination, an essential post-translational modification, plays a dual role in maintaining intracellular protein homeostasis and mediating intracellular signaling cascades. In their study of NFE2L3 function, Nouhi et al. (2007) discovered that NFE2L3 can undergo degradation via the ubiquitin-proteasome pathway. They identified two types of ubiquitination on NFE2L3 (Lys48 and Lys63) and highlighted K77 as a major ubiquitination site for NFE2L3 turnover facilitated by the E3 ligase F-box/WD repeat protein 7 (FBW 7) (Kannan et al., 2015). Chowdhury et al. (2017) revealed multiple ubiquitin-dependent degradation mechanisms for NFE2L3 at different cellular locations. For example, NFE2L3 can be rapidly degraded in the cytoplasm by the ER-associated ubiquitin ligase synoviolin (HRD1) and valosin containing protein (VCP), whereas in the nucleus, β-transducin repeat-containing protein (β-TRCP)-based E3 ubiquitin ligase mediates NFE2L3 degradation. Collectively, NFE2L3 ubiquitination occurs diverse forms within the cell and is targeted for degradation by various molecules, contributing to the regulation of NFE2L3 protein levels and signaling.

#### 3.2.3 Phosphorylation

In addition to glycosylation and ubiquitination modification, Kannan et al. (2015) discovered through an immunoprecipitation experiment that glycogen synthase kinase 3 (GSK3) can interact with NFE2L3. Furthermore, *in vitro* kinase assay revealed that the phosphorylation of NFE2L3 significantly increases when GSK3B and NFE2L3 are combined, compared to the control group. Notably, GSK3-mediated phosphorylation is a prerequisite for FBW7 to degrade NFE2L3 through ubiquitination.

**FIGURE 3:**
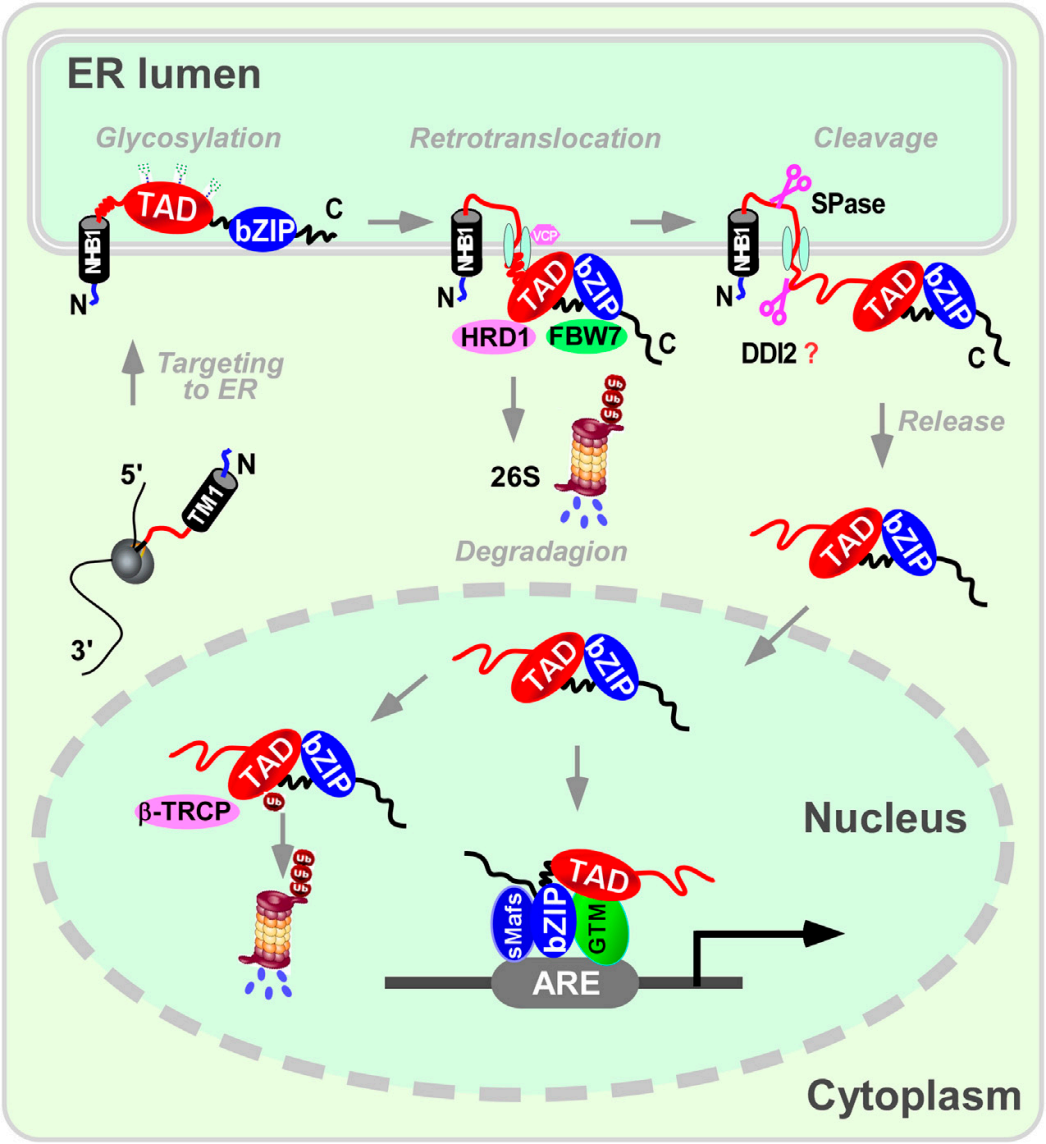
A proposed mechanism for NFE2L3 dynamic cleavage processing in endoplasmic reticulum. When anchored to the endoplasmic reticulum (ER) via signal peptides related to the N-terminal homology box 1 (NHB1) subdomain, the amino terminal of NFE2L3 orients to the cytoplasmic side while the carboxyl terminal locates in the ER lumen. Multiple modifications like glycosylation occur in the ER to produce the precursor of the mature protein. Upon specific stimulation, NFE2L3 is released from the ER with the aid of signal peptidase or DNA-damage inducible one homolog 2 (DDI2), then translocates to the nucleus for downstream gene regulation. Excess NFE2L3 is degraded through the ubiquitination-proteasome system in the cytoplasm and nucleus, involving ubiquitination-related proteins such as F-box/WD repeat protein 7 (FBW7), synoviolin/valosin containing protein (HRD1/VCP), and β-transducin repeat-containing protein (β-TRCP). It is important to note that there is no direct evidence showing DDI2 can cleave NFE2L3. ARE: antioxidant response element; bZIP: basic leucine-zipper; GTM: general transcriptional machineries; sMafs: small Maf; TAD: transactivation domain.

Taken together, NFE2L3 is structurally similar to NFE2L1 and undergoes similar post-translational modification processing (Yang et al., 2020) (Figures 2B, 3): when anchored to the ER via signal peptides related to the N-terminal homology box 1 (NHB1) subdomain, the amino terminal of NFE2L3 orients to the cytoplasmic side while the carboxyl terminal locates in the ER lumen (Zhang et al., 2009); multiple modifications such as glycosylation occur in the ER to produce the precursor of the mature protein; upon specific stimulation, NFE2L3 is released from the ER with the aid of signal peptidase or DNA-damage inducible one homolog 2 (DDI2), then translocates to the nucleus for downstream gene regulation. Excess NFE2L3 is degraded through the ubiquitination-proteasome system in the cytoplasm and nucleus, involving in ubiquitination-related proteins such as FBW7, HRD1/VCP, and β-TRCP (Chowdhury et al., 2017).

## 4 Biological function of NFE2L3

### 4.1 Role of NFE2L3 in regulating intracellular biological processes

#### 4.1.1 Regulation of NFE2L3 in oxidative stress

Wild-type mice treated with the antioxidant butylated hydroxytoluene (BTH) exhibited abnormal symptoms such as respiratory distress and weight loss, along with significant downregulation of *Nfe2l3* expression in the lungs. When *Nfe2l3* was knocked out in the entire body, mice became more sensitive to antioxidants, leading to acute lung injury and substantial weight loss (Chevillard et al., 2010). These findings indicate a crucial role for NFE2L3 in maintaining intracellular redox homeostasis. This notion has been further confirmed by several groups, such as silencing *Nfe2l3* in *Nfe2l2*-deficient keratinocytes, which inhibits the induction of antioxidant enzymes (heme oxygenase one and glutathione S-transferase Ya) in response to reactive oxygen species (ROS) inducers (Braun et al., 2002). However, Pepe et al. (2010) showed that overexpression of *Nfe2l3* could enhance intracellular ROS production during smooth muscle cell (SMC) differentiation. Chowdhury et al. (2009) revealed that the overexpression of *NFE2L3* significantly inhibited antioxidant oxidase *PRDX6* expression under oxidative stress. Notably, a dual-luciferase reporter assay showed that NFE2L3 can directly control the expression of NAD(P)H dehydrogenase, quinone 1 (*NQO1*) (Sankaranarayanan and Jaiswal, 2004). Interestingly, a contradictory outcome was observed in mouse *Nfe2l3*-overexpressed setting (Zhang et al., 2009), which may be attributed to differences in the length or species of the *NQO1* ARE site, or to disparities in the specific model cells utilized in their studies. These findings indicate that NFE2L3 plays a crucial role in preserving the intracellular redox balance. Nevertheless, the exact mechanism through which NFE2L3 regulates oxidative stress remains unclear.

#### 4.1.2 Regulation of NFE2L3 in proteostasis

Proteasomes are, large protein complexes found in eukaryotes and archaea that play crucial roles in the degradation of unfolded, damaged, or redundant proteins to maintain cellular homeostasis (Cockram et al., 2021). It is well documented that NFE2L1 has been identified as a key transcription factor that activates the expression of genes related to proteasomes, mitigating the effects of proteasome activity inhibition, known as the proteasome ‘bounce-back effect’ (Yang et al., 2020). Owing to its similarities to NFE2L1, NFE2L3 is speculated to be involved in the regulation of proteasome activity. In fact, a ChIP-seq experiment indeed found that NFE2L3 can directly bind to the ARE site in the promoter region of proteasome subunit genes (*PSMB3*, *PSMB7*, *PSMC2*, *PSMD3*, *PSMG3*, and *POMP*) (Waku et al., 2020a), and knockdown *NFE2L1* and *NFE2L3* simultaneously resulted in significantly downregulated proteasome activity, although no noticeable changes were observed in cells with individual knockdowns. Of note, knockdown *NFE2L3* showed a significant increase in the protein levels of NFE2L1 in colon cancer cells, implying a complex regulatory relationship between NFE2L3 and proteasome activity (Waku et al., 2020a). Further analysis revealed that when cells demonstrated a low level of NFE2L3, it could directly regulate the expression of proteasome subunit genes through elevating NFE2L1; when intracellular NFE2L3 was at a high level, it could not only directly regulate the expression of proteasome subunits, but also prevent *NFE2L1* translation through regulating its downstream gene cytoplasmic polyadenylation element binding protein 3 (CPEB3) to target the 3’ untranslated region of *NFE2L1* mRNA (Waku et al., 2020a). Meanwhile, another study from the same group revealed that NFE2L3 promotes the assembly of the 20S proteasome by directly inducing the expression of *POMP*, leading to accelerated degradation of tumor suppressor genes p53 and Rb (Waku et al., 2020b). These findings suggest that NFE2L3 directly regulates the expression of proteasome subunits and indirectly affects the translation of *NFE2L1*. The coordination between NFE2L3 and NFE2L1 plays an essential role in regulating proteasome function, ultimately contributing to the stable growth of tumor cells.

#### 4.1.3 Role of NFE2L3 in ER stress

Besides NFE2L3 regulates protein degradation via the proteasome, abnormal protein processing also affects the function of NFE2L3. This was evidenced by the time-dependent downregulation of all three NFE2L3 isoforms in tunicamycin (TU) or thapsigargin (TG)-induced ER stress in JAR cells (Nouhi et al., 2007). However, in contrast to these findings, Zhang et al. (2009) demonstrated that only the full glycosylation of NFE2L3 was decreased in response to the ER stressors TU and brefeldin A in *Nfe2l3*-overexpressed COS-1 cells, with the activation of its transcriptional activity, rather than TG. They proposed that these effects were not directly influenced by ER stress, but by glycosylation. Surprisingly, Gurri et al. (2023) recently reported that TU treatment increased the abundance of NFE2L3 in skin cancer SCC13 cells, and the knockdown of *NFE2L3* protected cancer cells from TU-induced apoptosis, possibly because of the stabilization of its partner, heat shock protein family A (Hsp70) member 5 (HSPA5). These results suggest that the role of NFE2L3 in ER stress is extremely complex and that we should carefully consider the different effects of experimental conditions, such as cell lines, treatment times, concentrations, and species.

#### 4.1.4 Regulation of NFE2L3 in lipid metabolism

Lipids, such as fatty acids, triglycerides, cholesterol, and phospholipids, play a significant role in maintaining the normal functioning of cellular processes (Xiang and Miao, 2021). As a transcription factor in the ER, NFE2L3 may play a role in lipid metabolism. This hypothesis is supported by the fact that the homologous protein NFE2L1 is essential for maintaining cholesterol homeostasis (Widenmaier et al., 2017). Actually, some findings from *Nfe2l3* knockout mice have revealed a connection between NFE2L3 and lipid metabolism, for example, there was a significant increase in the expression of the adipocyte differentiation gene peroxisome proliferator activated receptor γ in the white adipose tissue of *Nfe2l3* ^−/−^ mice, suggesting that NFE2L3 may be involved in the transcriptional regulation of this gene (Chevillard et al., 2010). Recently, Waku et al. (2021) discovered that NFE2L3 directly regulates genes associated with cholesterol synthesis. Their findings showed that NFE2L3 can either directly enhance the transcription of sterol regulatory element binding transcription factor 2 (SREBP2) or form a transcriptional complex with SREBP2 to boost the expression of mevalonate pathway genes such as 3-hydroxy-3-methylglutaryl-CoA synthase 1 (HMGCS1), 3-hydroxy-3-methylglutaryl-CoA reductase (HMGCR), and isopentenyl-diphosphate delta isomerase 1 (IDI1). Despite the overexpression of *NFE2L3*, intracellular cholesterol synthesis did not increase and the levels of its precursor lanosterol decreased. Further investigation revealed that NFE2L3 can convert the lanosterol precursor into a substantial amount of geranylgeranyl pyrophosphate to inhibit adipogenesis by upregulating geranylgeranyl diphosphate synthase 1 (GGPS1). Moreover, NFE2L3 directly enhances the transcription of Ras-related protein 5 (RAB5) to facilitate extracellular cholesterol uptake, thus ensuring intracellular cholesterol stability. In summary, these results demonstrated that NFE2L3 is crucial for intracellular lipid metabolism, especially for maintaining cholesterol homeostasis.

#### 4.1.5 Regulation of NFE2L3 in inflammatory response

KGF plays a vital role in the inflammatory process by reducing inflammation, exerting immunosuppressive effects, inhibiting the release of inflammatory mediators, and promoting skin healing. In a study on the role of NFE2L2 in wound healing, Braun et al. (2002) discovered that KGF triggered the expression of *Nfe2l3* in keratinocytes, and this increase was also noted in wounded skin, indicating the potential involvement of NFE2L3 in inflammation regulation. This was corroborated by the fact that colon inflammation was significantly reduced in *Nfe2l3* ^−/−^ mice (Saliba et al., 2022). Moreover, the transcription levels of the inflammatory factor interleukin 33 (Il33) were decreased in *Nfe2l3* knockout mice, and the mRNA and protein levels of ras-related protein Rab-27A (RAB27A), an important regulator of mast cells, were increased in mast cells obtained from azoxymethane (AOM) and dextran sodium sulfate (DSS) -induced *Nfe2l3* ^−/−^ mice, which resulted from the direct binding of NFE2L3 at the loci of *Il33* and *Rab27a*. Notably, the number of Tregs were increased in this model. Furthermore, analysis of RNA-seq data from the Human Microbiome Project (HMP2) showed that *NFE2L3* transcript levels were higher in the rectum of patients with ulcerative colitis (Saliba et al., 2022). Similarly, Chevillard et al. (2010) found that BTH can promote the expression of prostaglandinendoperoxide synthase 2 (*Ptgs2*), which was blocked by *Nfe2l3* knockout, thereby inducing an inflammatory response caused by lung injury in vitro animal experiments. It is worth mentioning that the inflammatory factor TNF-α can promote RELA to bind to the first intron of *NFE2L3* to activate its transcription, thereby affecting tumor progression (Bury et al., 2019). These results indicate that NFE2L3 participates in the regulation of inflammation. However, the precise regulatory mechanisms may vary temporally and spatially, necessitating further comprehensive investigation.

#### 4.1.6 Role of NFE2L3 in embryonic development and cell differentiation

Studies have found that *NFE2L3* is highly expressed in placental trophoblast cells (Chenais et al., 2005) and chicken mesoderm derivatives with spatio-temporal specificity (Etchevers, 2005). Interestingly, through the analysis of single-cell gene expression profiles from zygote to mid-gestation combined with siRNA library screening, *NFE2L3* was identified as one of the 15 hub genes involved in the self-renewal of human trophoblast stem cells (Chen Y. et al., 2022). These results strongly indicate that *NFE2L3* participates in mbryonic development, however, no significant differences were observed in growth, development, and fertility between *Nfe2l3* knockout mice and wild-type mice (Derjuga et al., 2004) and the expression of *NFE2L3* was also not changed during the process of thrombopoietin-induced megakaryocytic differentiation of CD34^+^ cells (Terui et al., 2000). Nevertheless, two *in vitro* experiments showed that NFE2L3 promotes the differentiation of embryonic stem cells into SMC by directly regulating the expression of the SMC transcription factors *Myocardin* (Pepe et al., 2010) and phospholipase A2 group VII (Pla2g7) (Xiao et al., 2012). It is remarkable that to investigate the potential functional redundancy between NFE2L3 and other CNC subfamily members in growth and development, Derjuga et al. (2004) generated *Nfe2l3* ^−/−^/*Nfe2l2* ^−/−^ and *Nfe2l3* ^−/−^/*p45* ^−/−^ knockout mice, and found both of which exhibited normal growth. This phenomenon implies that the deficiency in NFE2L3 function may be functionally compensated for by its homologous protein or other unknown proteins.

**FIGURE 4:**
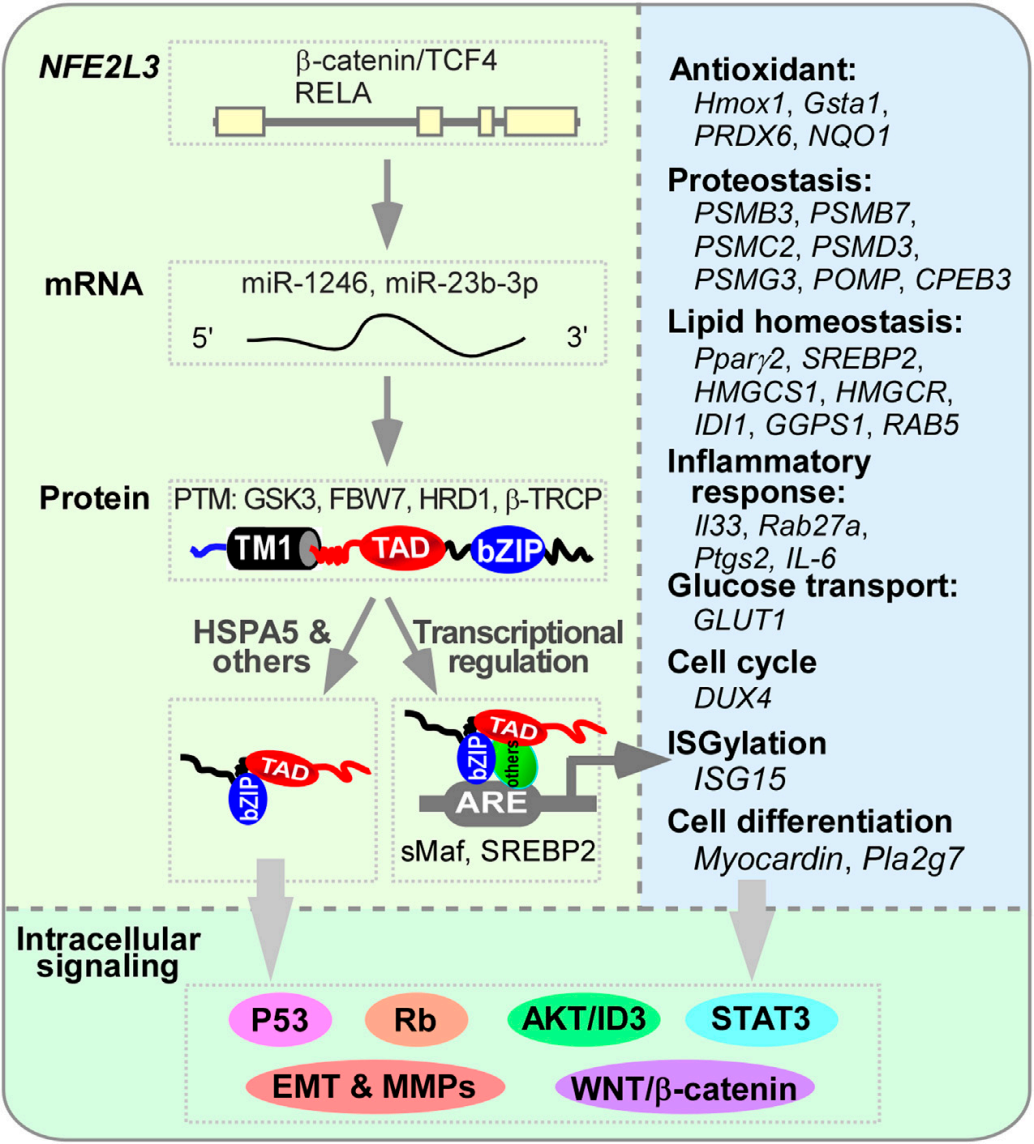
The regulation of NFE2L3 and its downstream genes. The transcription of *NFE2L3*, which consists of four exons, is activated by the β-catenin/TCF4 complex or RELA (RELA proto-oncogene, NF-kB subunit) through binding to its first intron. This activation is further regulated by microRNAs such as miR-1246 and miR-23b-3p. Following translation, NFE2L3 located in the endoplasmic reticulum (ER) undergoes post-translational modifications like phosphorylation and ubiquitination by GSK3, FBW7, HRD1/VCP, and β-TRCP before maturing into a protein. Once released from the ER, NFE2L3 can translocate into the nucleus and bind to sMaf or SREBP2 to activate the expression of various genes involved in processes of antioxidant, proteostasis, lipid homeostasis, inflammatory responses, glucose transport, cell cycle regulation, ISGylation, and cell differentiation. Through direct regulation of these gene expressions or binding to HSPA5 or other unidentified factors, NFE2L3 can modulate multiple intracellular signaling pathways such as p53, Rb, STAT3, EMT, MMPs, AKT/ID3, and Wnt/β-catenin, thereby influencing cellular activities.

Besides its involvement in the processes of oxidative stress, proteostasis, ER stress, lipid metabolism, inflammatory response, and cell differentiation (Figure 4), NFE2L3 was also found to participate in the regulation of cell adhesion (Siegenthaler et al., 2018), neuroprotection and long-distance axon regeneration (Lukomska et al., 2024), and other undetermined processes, such as stress granule assembly, extracellular exosomes, cellular iron ion homeostasis, and autolysosomes (Liu et al., 2019).

### 4.2 Role of NFE2L3 in human disease

Given its importance in regulating intracellular redox balance, protein and lipid homeostasis, and cell differentiation, abnormal expression of NFE2L3 is strongly associated with both normal bodily functions and the development of various tumors (Table 2), which was elucidated as follows.

**TABLE 2:** The role of NFE2L3 in human cancers.

Tumor type	Object of study	Intervention	Biological function	Clinical relevance	References
Thyroid cancer	Cell lines (FTC236, and HTh74); The Cancer Genome Atlas (TCGA) dataset	siRNA, Overexpression	Knockdown of *NFE2L3* decreases the spheroid formation, growth, and invasiveness of thyroid cancer cells	*NFE2L3* is elevated in thyroid cancer samples and in distant metastasis samples	Wang et al. (2017)
	Papillary thyroid tumor samples	—	—	*NFE2L3* expression is higher in papillary thyroid cancer samples	Khanal et al. (2023)
Colorectal cancer	Cell lines (DLD-1, and HCT116)	siRNA	Silencing *NFE2L3* triggers cell cycle arrest (G0/G1), and declines cell proliferation	—	Chowdhury et al. (2017)
	Gene Expression Omnibus (GEO) datasets (GSE32323, GSE74602, and GSE113513) and TCGA	—	—	*NFE2L3* is identified as one of nine prognostic gene for diagnosis and prognosis prediction of colorectal cancer patients	Chen et al. (2019)
	GEO (GSE31279, GSE35602, and GSE46824), TCGA, SurvExpress (colon metabase)	—	—	*NFE2L3* is identified as a colon tumor stroma-specific transcriptional gene	Uddin et al. (2019)
	Cell lines (HCT116 and HT29); Female athymic nu/nu mice; TCGA, BioGPS and Oncomine datasets; Clinical samples	shRNA	*NFE2L3* transcripts were upregulated in colon adenocarcinoma; Silencing *NFE2L3* inhibits colon cancer cell proliferation and tumor growth in mouse xenograft model	Upregulation of *NFE2L3* correlates with poor prognosis for colon cancer	Bury et al. (2019)
	Cell line (HCT116) *Apc* ^flox/flox^ and *Apc* ^Δ716^ mice	siRNA	Silencing *NFE2L3* significantly reduced cell proliferation; *Apc* gene deletion induces *Nfe2l3* expression in mouse intestine and organoids	*NFE2L3* expression is induced in colon and rectal carcinoma	Aono et al. (2019)
	Cell lines (HCT116 and SW480) UCSC Cancer Browser and TCGA; Clinical samples	siRNA	*NFE2L3* knockdown arrests cell cycle at the G0/G1 phase through downregulation of CCND1 and pRb1-ser^807/811^	*NFE2L3* is markedly upregulated in colorectal cancer	Zhang et al. (2019)
	Cell lines (HCT116 and H1299); BALB/cA-nu female mouse	siRNA, Overexpression	*NFE2L3* knockdown significantly inhibited the growth of cancer cells; *NFE2L3* overexpression increased 20S proteasome activity via increase *POMP*; *NFE2L3* overexpression decreases Rb and p53 protein through ubiquitin-dependent degradation; Overexpression of *NFE2L3* induced tumor growth and hepatic metastasis	Higher *NFE2L3* expression is correlated with poor prognoses in colorectal or rectal adenocarcinoma patients	Waku et al. (2020b)
	Cell lines (NCM460, SW620, SW1116, CW-2, and LoVo) TCGA	Overexpression	miR-23b-3p inhibited the proliferation, migration and invasion of colon adenocarcinoma cells by directly downregulating *NFE2L3*	*NFE2L3* expression is elevated in colon adenocarcinoma at different stages	Huang et al. (2021)
	Cell line (HCT116); *Nfe2l3* ^−/−^ mice; HMP2 datasets	AOM (7 mg/kg)/DSS (2.5%) treatment	*Nfe2l3* ^−/−^ mice exhibit significantly less inflammation in the colon, reduced tumor size and numbers; NFE2L3 deficiency disrupts mast cell homeostasis via downregulation of *Il33* and *Rab27a*	*NFE2L3* transcript levels are higher in the rectum of ulcerative colitis patients, compared to these in normal groups	Saliba et al. (2022)
Breast cancer	TCGA; Clinical samples	—	—	Hypermethylated *NFE2L3* is associated with invasive breast cancer; *NFE2L3* displays hypermethylation for estrogen receptor positive tumors and hypomethylation for estrogen receptor negative tumors	Rauscher et al. (2015)
	Cell lines (MCF-7, SKBR3, and MDA-MB-231)	siRNA, Overexpression	NFE2L3 inhibited breast cancer cell proliferation and migration by inhibiting AKT/ID3 axis; Silencing NFE2L3 increased the percentage of cell number in S and G2/M phase, and decreased these in G0/G1 phase	*NFE2L3* is positively related to the survival of breast cancer patients	Sun et al. (2019)
	Cell lines (MDA-MB-231 and SKBR3)	siRNA	Inhibition of miR-1246 increases *NFE2L3* expression, which may contribute to increase the sensitivity of cells to docetaxel and decrease the ability of cell migration	—	Dai et al. (2021)
	Cell lines (BT-549, MDA-MB-231, and HCC-70); Sequence Read Archive database	siRNA	Knockdown of *NFE2L3* inhibits colony formation of triple-negative breast cancer (TNBC) models, and enhances the sensitivity of paclitaxel	*NFE2L3* is upregulated in **TNBC** cancer but not enriched in any TNBC subsets	Elango et al. (2021)
Hepatocellular carcinoma	Cell lines (SMCC-7721 and BEL7404); TCGA	shRNA	Knockdown of *NFE2L3* inhibits cell proliferation, induces cell apoptosis, and suppresses the migration, invasion, and EMT of hepatocellular carcinoma cell	*NFE2L3* positively correlated with tumor grade, T stage, and pathologic stage	Yu et al. (2019)
	Cell line (HepG2); GEO (GSE25097, and GSE76427) and TCGA	shRNA	Knockdown of *NFE2L3* inhibits cell proliferation and migration, arrests cell cycle at G0/G1 phase and induces cell apoptosis, thereby inhibiting the malignant growth of subcutaneous carcinoma xenograft. Deficiency of *NFE2L3* decrease the process of EMT.	*NFE2L3* expression is upregulated and associated with hepatocellular carcinoma	Ren et al. (2020a)
	Cell lines (HepG2, MHCC97H); Nude mice; TCGA; Tissue microarrays and clinical samples	siRNA, shRNA	Overexpression of *NFE2L3* promotes hepatocellular carcinoma cell proliferation; NFE2L3 enhances p53 degradation	*NFE2L3* is associated with overall survival in hepatocellular carcinoma	Ren et al. (2023)
Gastric cancer	TCGA	—	—	The methylation of *NFE2L3* is associated with gastric cancer	Peng et al. (2020)
	Cell lines (SGC-7901 and MGC803); GEO (GSE103236) and TCGA	shRNA	Inhibiting *NFE2L3* expression blocks the cell cycle at G0/G1 phage, induces cell apoptosis, and decreases biomarkers in EMT.	*NFE2L3* is upregulated in gastric cancer patients, with a shorter survival time	Wang et al. (2021)
	TCGA, UCSC Xena, Human Protein Atlas; Clinical samples	siRNA, overexpression	Knockdown of *NFE2L3* inhibits the proliferation and migration of cancer cells; *vice versa*	*NFE2L3* is elevated in cancer, and high levels of *NFE2L3* are associated with poor overall survival, progress-free interval, and disease-specific survival	Li and Wen (2024)
Renal cancer	GEO (GSE70303, GSE113501, GSE6344 and GSE53757) and TCGA	—	—	The methylation of *NFE2L3* is decreased in tumor tissue; DNA methylation-driven *NFE2L3* may be a prognostic marker in human clear cell renal cell carcinoma	Wang et al. (2019)
	GEO (GSE29609, GSE40435, GSE53757, and GSE70303), and TCGA	—	—	DNA methylation-driven *NFE2L3* may be a prognostic marker in human clear cell renal cell carcinoma	Zhang et al. (2020)
	Cell lines (LoMet-ccRCC, RPTEC, and Caki-1); TCGA	5-Aza-CdR (0–10 μM), shRNA, Overexpression	*NFE2L3* overexpression increases cancer cell proliferation, migration, and invasion	The methylation levels of *NFE2L3* are decreased in tumor tissues, with an increase in *NFE2L3* mRNA levels, which is negatively correlated to survival time of patients	Hou et al. (2024)
Pancreatic cancer	Cell lines (PANC-1 and SW 1990); GEO (GSE16515, GSE15471, GSE55643, and GSE28735); TCGA; Clinical smaples	siRNA	Silencing *NFE2L3* inhibits cell invasion ability, whereas cell proliferation demonstrates no obvious changes	High levels of *NFE2L3* are associated with the poor prognosis of pancreatic cancer patients	Wang et al. (2018)
	GEO (GSE15471), TCGA, and OncoLnc; Clinical samples	—	—	*NFE2L3* is an independent prognostic factors for short patient survival times in pancreatic ductal adenocarcinoma	Tanaka et al. (2020)
Bladder cancer	Cell lines (RT4, BIU-87, J82, EJ, UM-UC-3, 5637, T24, and SW780); GEO (GSE40355, GSE12507, GSE37815, GSE32548, and GSE19915), and UCSC Xena	shRNA, Overexpression	Knockdown of *NFE2L3* inhibits cells proliferation, arrests cell cycle, and induces cell apoptosis; Overexpression of *NFE2L3* promotes cell migration and invasion, *in vitro* and *in vivo*, and EMT.	*NFE2L3* is increased in bladder cancer samples, which is associated with poor clinical outcomes	Qian et al. (2022)
Squamous cell carcinomas	Cell lines (KYSE-150 and ECA-109R); GEO (GSE53625) and TCGA	shRNA, Overexpression	Cells proliferation and migration is promoted by *NFE2L3* overexpression, and is inhibited by silencing *NFE2L3*; NFE2L3 increased radioresistance in esophageal squamous cell carcinoma cells	A significant upregulation of *NFE2L3* expression was documented in esophageal cancer	Chen et al. (2022b)
	Cell lines (SCC13 and HaCaT); *Nfe2l3* ^−/−^ mice and NOD-SCID mice; Clinical samples	7,12-dimethylbenz(a)anthracene (100 μg in 300 μL acetone), 12-O-tetradecanoylphorbol-13-acetate (15 lg in 200 ll acetone), siRNA, CRISPR/Cas9-ko, Lentivirus (Overexpression)	Knockdown of *Nfe2l3* promotes growth and malignant conversion of chemically induced skin tumors; Loss of NFE2L3 promotes clonogenicity and migration of the cancer cells, enhances tumorigenesis and invasiveness	NFE2L3 is downregulated at the protein level in invasively growing skin cancer cells	Gurri et al. (2023)
T-cell lymphoblastic lymphoma	*Nfe2l3* ^−/−^ mice	benzo-[a]pyrene (B[a]P) (100 mg/kg)	*Nfe2l3* ^−/−^ mice exhibits highly susceptible to B[a]P, with a high incidence of T-cell lymphoblastic lymphoma, and demonstrates significantly increased mortality	—	Chevillard et al. (2011)
Lung cancer	GEO (GSE72094), and TCGA	—	—	Methylation driven *NFE2L3* is correlated with lung adenocarcinoma prognosis	Ren et al. (2020b)
Larynx carcinoma	Cell line (TU686)	—	APOM overexpression increases the mRNA level of *NFE2L3*, with no changes in protein level	—	Xue et al. (2020)

#### 4.2.1 Role of NFE2L3 in tumor progression

##### 4.2.1.1 Thyroid cancer

When analyzing The Cancer Genome Atlas (TCGA) database, Wang et al. (2017) discovered a significant increase in the expression level of *NFE2L3* in human thyroid cancer tissues compared to that in normal tissues. This finding was further validated by qPCR experiments in another study (Khanal et al., 2023). Subsequent gene chip screening and experimental analysis indicated that the downregulation of RCAN1-4 could enhance the growth and metastasis of thyroid cancer cells by upregulating NFE2L3 expression (Wang et al., 2017). In general, although both *in vivo* and *in vitro* experimental and clinical evidence suggest that inhibiting NFE2L3 expression may impede the progression of thyroid cancer, the precise mechanism by which NFE2L3 exerts its pro-tumor effects remains unclear.

##### 4.2.1.2 Colorectal cancer

Recent studies have shown that *NFE2L3* is increased in colorectal cancer and has a positive correlation between *NFE2L3* expression in tumor grade and stage (Bury et al., 2019; Chen et al., 2019; Uddin et al., 2019; Liu et al., 2022). Both *in vitro* and *in vivo* experiments have demonstrated that knocking down *NFE2L3* leads to a reduction in the proliferation of colon cancer cells, thereby inhibiting tumor growth (Chowdhury et al., 2017; Bury et al., 2019). These findings are further supported by studies showing that modulation *NFE2L3* with miR23b-3p can reproduce similar results (Huang et al., 2021). Notably, one of the key mechanisms by which NFE2L3 exerts its tumor-promoting effects is the regulation of cell cycle progression. Bury et al. (2019) demonstrated that NFE2L3 promotes the proliferation of colon cancer cells by transcriptionally inhibiting the expression of DUX4, a molecule that inhibits cyclin CDK1. Moreover, following *NFE2L3* knockdown, the cell cycle regulatory factors UHMK1, CCND1, and pRb1-Ser^807/811^ are downregulated in colon cancer cells, leading to cell cycle arrest at the G0/G1 phase (Chowdhury et al., 2017; Zhang et al., 2019). In addition to regulating cell cycle-related proteins, NFE2L3 can also affect cell activity by enhancing the degradation of tumor suppressor genes such as p53 and Rb by increasing the function of the 20S proteasome (Waku et al., 2020b). In addition, Saliba et al. (2022) found a significant reduction in both the size and number of colon tumors in *Nfe2l3* ^−/−^ mice, along with weakened colon inflammation, compared to wild-type mice in an inflammation-induced colon cancer model (AOM/DSS). Subsequent RNA-seq analysis revealed that only mast cells showing significant changes in tumor tissues, that is, activated mast cells were predominant in wild-type mouse tumors, whereas resting mast cells were more prevalent in *Nfe2l3* ^−/−^ mice. Moreover, digital spatial profiling and immunohistochemistry demonstrated that *Nfe2l3* ^−/−^ mice promote the infiltration of tumor-suppressive Tregs, ultimately leading to an immunosuppressive tumor microenvironment (Saliba et al., 2022). Despite demonstrating the impact of *Nfe2l3* ^−/−^ on mast cell function through *Il33* and *Rab27a*, this study utilized systemic gene knockout mice, leaving out the specific cellular and regulatory mechanisms underlying the creation of an immunosuppressive tumor microenvironment by NFE2L3 deletion.

Additionally, Aono et al. (2019) discovered that the β-catenin/TCF4 complex directly regulates *NFE2L3* expression, and both *β-catenin/TCF4* and *Nfe2l3* are significantly activated in a spontaneous colon cancer model resulting from *APC* gene mutation. Of note, one reason for the carcinogenesis of this mutation was the global cellular metabolic reprogramming mediated by the abnormal expression of protooncogenes such as β-catenin. These findings, together with the fact that abnormal expression of NFE2L3 can also lead to metabolic reprogramming by affecting the expression of glucose transporter *GLUT1* in colorectal cancer cells (Aono et al., 2019) and *Nfe2l3* was identified as one of the five most variable genes in the AOM/DSS model (Suzuki et al., 2007), suggest that NFE2L3 may play a crucial role in the early stages of colon cancer development, although distinct phenotypes were not observed in *Nfe2l3* knockout mice. In conclusion, NFE2L3 could serve as a valuable biomarker or therapeutic target in the context of colorectal cancer.

##### 4.2.1.3 Breast cancer

Breast cancer is the second most common cause of cancer-related death in women (Sung et al., 2021). Sun et al. (2019) revealed that the expression of *NFE2L3* in breast cancer tissues was significantly reduced and was negatively correlated with lymph node metastasis and tumor stage. Furthermore, silencing *NFE2L3* increased MCF-7 cell cycle progression and enhanced cell proliferation, whereas overexpression of *NFE2L3* effectively restricted the growth and metastasis of cancer cells and inhibited the epithelial-mesenchymal transformation (EMT) and MMPs expression. Additionally, activated NFE2L3 can prevent the malignant progression of breast cancer by inhibiting the AKT/ID3 axis (Sun et al., 2019). This mechanism was further confirmed by Dai et al. (2021), who found that miR-1246 inhibits the activation of AKT/ID3 axis by targeting *NFE2L3* mRNA, thus promoting drug resistance and metastasis of breast cancer. However, Elango et al. (2021) showed that the knockdown of NFE2L3 significantly inhibits colony formation in triple-negative breast cancer cells. These contradictory results may be related to the discrepancy in the methylation level of *NFE2L3* in different types of breast cancer cells (Rauscher et al., 2015), which requires further verification.

##### 4.2.1.4 Hepatocellular carcinoma

In contrast to breast cancer, Liu et al. (2022) analyzed an RNA-sequencing database and found that the expression of *NFE2L3* in liver cancer was positively correlated with tumor grade and stage, and analysis of clinical patient tissue samples also revealed a significant association between high *NFE2L3* levels and poor prognosis in patients with liver cancer. *In vitro* experiments have demonstrated that suppression of *NFE2L3* inhibits cell proliferation, migration, invasion, and EMT, ultimately leading to apoptosis in liver cancer cells (Yu et al., 2019; Ren Y. et al., 2020). Ren Y. et al. (2020) also noted a positive correlation between *NFE2L3* expression and the aggressive behavior of liver cancer cells and EMT. This oncogenic function of NFE2L3 is likely attributed to its downregulation of cyclin D1 and TCF4 by inhibiting the Wnt/β-catenin signaling pathway. Recently, another study by this group uncovered a new role for NFE2L3 in promoting liver cancer (Ren et al., 2023). NFE2L3 upregulates the expression of interferon-stimulated gene 15 (ISG15), leading to the ISG modification of the p53. The modified p53 protein is rapidly degraded by NFE2L3-regulated proteasomes, ultimately enhancing the malignant capabilities of liver cancer cells.

In summary, these results indicate that NFE2L3 plays an important role in regulating the occurrence, development, and prognosis of hepatocellular carcinoma. Therefore, targeting NFE2L3 may be an important strategy for the treatment of hepatocellular carcinoma.

##### 4.2.1.5 Gastric cancer

Gastric cancer is a prevalent gastrointestinal malignancy with an incidence of 5.6% and a mortality rate of 7.7%, ranking fifth and fourth among all cancer types, respectively (Sung et al., 2021). Owing to the limitations of the current TNM staging system in gastric cancer, Peng et al. (2020) identified new biomarkers for the diagnosis, prognosis, and prediction of gastric cancer. They analyzed the DNA methylation characteristics of gastric cancer using public databases and identified 10 candidate genes, including *NFE2L3*, that were associated with gastric cancer recurrence. Subsequent research by Wang et al. (2021) confirmed these findings by analyzing clinical samples and common tumor cell lines. They observed that *NFE2L3* expression in gastric cancer tissues was significantly higher than that in the adjacent tissues. Moreover, the knockdown of *NFE2L3* led to inhibited cellular biological behaviors such as proliferation, migration, and invasion of gastric cancer cells, along with cell cycle arrest and increased apoptosis (Wang et al., 2021; Li and Wen, 2024). Further studies revealed that E-cadherin expression was upregulated, whereas vimentin and N-cadherin expression were downregulated, suggesting a possible connection between NFE2L3 and EMT in gastric cancer. However, the underlying regulatory mechanisms remain unclear.

##### 4.2.1.6 Renal cancer

Current research on NFE2L3 in renal cancer has primarily focused on analyzing sequencing data (Wang et al., 2019; Zhang et al., 2020; Bai et al., 2021; Zhang et al., 2022). Findings showed that *NFE2L3* expression was higher in clear cell carcinoma of the kidney (KIRC) than in normal tissue. This trend has also been observed in renal papillary cell carcinoma (Zhang et al., 2022), suggesting a potential role for *NFE2L3* in renal cancer progression. Additionally, these data suggest that NFE2L3 may affect the tumor immune microenvironment by influencing antigen processing and presentation, NOD-like receptor signaling pathway, Toll-like receptor signaling pathway, lymphocyte-mediated immune regulation, and adaptive immune response, thereby contributing to the development of renal clear cell carcinoma (Wang et al., 2019; Zhang et al., 2022). Furthermore, Bai et al. (2021) identified NFE2L3 as one of the six genes significantly associated with survival differences in KIRC in a hypoxia-immune-related prognostic risk model. Importantly, through the analysis of multiple databases, *NFE2L3* was found to be strongly correlated with the prognosis of DNA methylation-driven KIRC (Wang et al., 2019; Zhang et al., 2020), indicating its potential as a prognostic biomarker for renal cancer. Notably, the tumor-promoting effect of NFE2L3 in renal cancer was recently confirmed *in vitro* experiments (Hou et al., 2024) and our ongoing work. However, the molecular mechanisms underlying the action of NFE2L3 in the pathogenesis and progression of renal cancer remain largely unknown.

##### 4.2.1.7 Bladder cancer

Qian et al. (2022) demonstrated a significant increase in *NFE2L3* expression in bladder cancer (BLCA) samples compared to normal tissues and found a strong correlation between high levels of *NFE2L3* and advanced clinicopathological features, as well as poor prognosis. Importantly, intervention in *NFE2L3* expression suppressed the progression of BLCA, potentially through the regulation of NFE2L3 in the cell cycle, apoptosis, and EMT (Qian et al., 2022), indicating that NFE2L3 is involved in the advancement of BLCA. These findings imply that NFE2L3 could serve as a crucial biomarker and potential therapeutic target for predicting clinical outcomes in BLCA. However, *Nfe2l3* knockout mice-based BLCA models must be used to validate these results.

##### 4.2.1.8 Pancreatic cancer

By analyzing various datasets, cell lines, and clinical samples, Wang et al. (2018) discovered that *NFE2L3* levels in pancreatic cancer tissues were elevated compared to those in normal tissues at both the mRNA and protein levels. Additionally, clinical data indicated a strong correlation between high *Nfe2l3* expression and lymph node metastasis, advanced TNM stage, and poor prognosis, which may be attributed to alterations in VEGFA. In addition, NFE2L3 also was identified as an independent prognostic factor for the survival of patients with pancreatic ductal adenocarcinoma (Tanaka et al., 2020).

##### 4.2.1.9 Squamous cell carcinoma

It is reported that *Nfe2l3* participates in the normal healing of skin cells and potentially compensates for the loss of NFE2L2 (Braun et al., 2002), highlighting the importance of NFE2L3 in the regulation of skin function. However, no healing abnormalities were observed in *Nfe2l3* ^−/−^ mice with full-thickness excisional wounds compared with wild-type mice (Siegenthaler et al., 2018). Surprisingly, the deficiency of *Nfe2l3* protects keratinocytes from UVB-, oxidative-, and hyperosmotic stress-induced apoptosis by activating cell adhesion signals. Recently, Gurri et al. (2023) demonstrated that the NFE2L3 protein level was decreased in human non-melanoma skin cancer. Moreover, knockout of *NFE2L3* promotes the growth and malignant conversion of squamous cell carcinomas induced by 7,12-dimethylbenzo(a)anthracene and 12-O-tetradecanoylphorbol-13-acetate. This conclusion was further confirmed in multiple models, such as ear tumorigenicity assay, UVB irradiation, and organotypic skin cultures. Taken together, these results strongly imply that NFE2L3 plays a protective role against skin cells, especially during tumor growth. Notably, a contrasting role of NFE2L3 was observed in multiple esophageal squamous cell carcinoma cell lines, and *in vitro* and *in vivo* experiments showed that the knockdown *NFE2L3* enhanced the radiosensitivity of esophageal squamous cell cancer through the transcriptional regulation of IL-6-mediated STAT3 signaling (Chen T. et al., 2022). These findings suggest that the role of NFE2L3 in squamous cell carcinoma may be tissue specific, and requires further investigation.

##### 4.2.1.10 T cell lymphoblastic lymphoma

In a study investigating NFE2L3 function, Chevillard et al. (2011) exposed *Nfe2l3* ^−/−^ mice to the carcinogen B[a]P (benzo[a]pyrene), and observed that only one out of 16 wild-type mice died before the 30th week, whereas six out of 19 (32%) *Nfe2l3* ^−/−^ mice died starting at week 15 after B[a]P treatment. This indicated that *Nfe2l3* ^−/−^ mice exhibited increased sensitivity to carcinogen exposure. Furthermore, following B[a]P treatment, 6% of the wild-type mice developed lymphoma, in contrast to 32% of *Nfe2l3* ^−/−^ mice. These findings suggest a potential protective role for NFE2L3 in the development of hematopoietic malignancies, warranting further identification of the underlying mechanisms.

In addition to the aforementioned studies, which have been verified through *in vitro* experiments, there are reports based on RNA sequencing data analysis indicating a potential correlation between abnormal *NFE2L3* expression and the prognosis of various cancers, such as lung adenocarcinoma (Ren J. et al., 2020), malignant pleural mesothelioma (Wang et al., 2022), ovarian cancer (Dou et al., 2022), glioblastoma multiforme (Rowther et al., 2016), and laryngeal carcinoma (Xue et al., 2020). Nevertheless, it is essential to validate these findings by modulating the expression of NFE2L3.

#### 4.2.2 Role of NFE2L3 in other diseases

Recent studies utilizing GWAS, microarray, transcriptome sequencing, and other technologies have identified a strong association between *NFE2L3* and other diseases, such as endometriosis (Painter et al., 2011; Osinski et al., 2018; Cardoso et al., 2020), gestational diabetes mellitus (Yan et al., 2018), chronic sinusitis (Ball et al., 2020), idiopathic pulmonary hypertension (Li et al., 2023), myopia (Simpson et al., 2021), diabetic foot ulcers (Jin et al., 2024), and obesity-related body fat distribution (Heid et al., 2010; Monda et al., 2013). However, further animal studies are required to elucidate the role of NFE2L3 in the development of these diseases.

## 5 Conclusion and future perspective

The structure and biological function of NFE2L3 suggest that it plays a role in various cellular processes, such as oxidative stress, the inflammatory response, lipid homeostasis, proteostasis, and cell differentiation, as a member of the CNC-bZIP subfamily. Dysregulation of NFE2L3 is closely linked to the development of various diseases, particularly tumors. Thus, targeted modulation of NFE2L3 is crucial for preserving normal cellular function. Current research on NFE2L3 is in its early stages, with many aspects yet to be explored. One key question is why NFE2L3 exhibits different functions in various tissues and organs, particularly during tumor progression. For instance, although highly expressed NFE2L3 promotes cancer in most tumors, it inhibits tumor progression in T cell lymphoblastic lymphoma, skin squamous cell carcinoma, and some breast cancers. Moreover, given the similarity between NFE2L3 and NFE2L1, whether NFE2L3 is a receptor of ER stress like NFE2L1 remains to be clarified. Notably, NFE2L3 not only regulates proteasome-mediated protein degradation, but also participates in the regulation of signals related to glucose metabolism (Aono et al., 2019) and cholesterol synthesis (Waku et al., 2021). These findings suggest that NFE2L3, similar to NFE2L1, is a crucial protein involved in intracellular glucose, lipid, and protein metabolism. However, the mechanism by which these two proteins collaborate to maintain the balance between these three major nutrients within cells remains unknown. Furthermore, bioinformatics analysis revealed a close connection between abnormal NFE2L3 function and changes in the tumor microenvironment. However, the impact of abnormal NFE2L3 expression in tumor cells on the tumor immune microenvironment and the specific role of NFE2L3 in immune cells remain unclear, although it was identified as a crucial gene involving in the stimulation of innate immune activity (Fairfax et al., 2014). It is noteworthy that previous research on NFE2L3 in tumor-related studies has shown a significant correlation with poor prognosis in various diseases, and modulating NFE2L3 expression can potentially impede tumor progression, highlighting NFE2L3 as a promising target for tumor treatment. Therefore, the identification of drugs targeting NFE2L3 is crucial for advancing tumor therapy. Importantly, present studies have made the targeting of transcription factors in cancer a reality (Bushweller, 2019), and clinical trials have shown that transcription factors are prospective therapeutic targets and reliable biomarkers for cancer diagnosis and prognosis (Silva et al., 2024), offering promising avenues for the development of novel transcription factor-based strategies in cancer treatment. Moreover, the development of chemicals targeting or regulating the activity of NFE2L1 and NFE2L2, two closely-related transcription factors of NFE2L3, shows significant potential to overcome chemotherapy drug resistance (Yuan et al., 2018; Jia et al., 2022) and inhibit tumor cell growth (Robledinos-Anton et al., 2019). Therefore, given the pivotal role of NFE2L3 in cancer, we firmly believe that advancing NFE2L3-targeted therapies will greatly improve cancer treatment.
